# Nanostructure-Mediated Transport of Therapeutics through Epithelial Barriers

**DOI:** 10.3390/ijms25137098

**Published:** 2024-06-28

**Authors:** M. Eva Hansen, Yasmin Ibrahim, Tejal A. Desai, Michael Koval

**Affiliations:** 1 University of California Berkeley-University of California San Francisco Graduate Program in Bioengineering, San Francisco, CA 94143, USA; eva.hansen@berkeley.edu; 2 Department of Bioengineering and Therapeutic Sciences, University of California San Francisco, San Francisco, CA 94143, USA; 3 Division of Pulmonary, Allergy, Critical Care and Sleep Medicine, Department of Medicine, Emory University School of Medicine, Atlanta, GA 30322, USA; yasmin.ibrahim@emory.edu; 4 Graduate Program in Biochemistry, Cell and Developmental Biology, Graduate Division of Biological and Biomedical Sciences, Emory University, Atlanta, GA 30322, USA; 5 School of Engineering, Brown University, Providence, RI 02912, USA; 6 Department of Cell Biology, Emory University School of Medicine, Atlanta, GA 30322, USA

**Keywords:** tight junctions, barrier permeability, drug delivery, transcytosis

## Abstract

The ability to precisely treat human disease is facilitated by the sophisticated design of pharmacologic agents. Nanotechnology has emerged as a valuable approach to creating vehicles that can specifically target organ systems, effectively traverse epithelial barriers, and protect agents from premature degradation. In this review, we discuss the molecular basis for epithelial barrier function, focusing on tight junctions, and describe different pathways that drugs can use to cross barrier-forming tissue, including the paracellular route and transcytosis. Unique features of drug delivery applied to different organ systems are addressed: transdermal, ocular, pulmonary, and oral delivery. We also discuss how design elements of different nanoscale systems, such as composition and nanostructured architecture, can be used to specifically enhance transepithelial delivery. The ability to tailor nanoscale drug delivery vehicles to leverage epithelial barrier biology is an emerging theme in the pursuit of facilitating the efficacious delivery of pharmacologic agents.

## 1. Introduction

Epithelial barriers serve as a boundary to separate external from internal microenvironments throughout the body. As such, barrier function is characteristic of epithelial tissues across many organ systems, including the skin, eyes, gastrointestinal tract, and lungs. Epithelial barriers are not absolute; instead, they vary depending on tissue type to allow selective solute permeability [1,2,3]. Net epithelial barrier function is due to the combined function of mucosal or cornified layers, transcellular barriers, and paracellular barriers, which together serve to restrict the passage of molecules and solutes from external environments and maintain tissue homeostasis [4,5]. Epithelial barriers, therefore, present an important design consideration when developing strategies for therapeutic delivery.

One approach to bypass epithelial barriers is subcutaneous or intravenous injection of therapeutics, but bolus administration via these routes presents limited control over therapeutic targeting and distribution. Thus, traversing epithelial barrier tissue in a controlled manner remains a prominent challenge for drug delivery, especially when considering therapeutic absorption and bioavailability of protein biologics and peptides. This has motivated extensive research to engineer particles and devices with nano- and microtechnologies to enable therapeutics to transit from the external or luminal environment to reach targets.

Pharmacologic approaches to promote transepithelial permeability typically leverage substances or surfaces directly interacting with barrier-forming cells, causing a physiologic response to enhance permeability. This includes integrated systems that contain therapeutic cargo or adjuncts that facilitate the delivery of an independently administered therapeutic agent.

Engineered nanostructures encompass a variety of form factors which impart distinct benefits in designing successful therapeutic delivery. Material selection is particularly critical in that it can be engineered to contain nanoarchitecture and/or enable surface modification to tailor cell interactions, control release, and protect the therapeutic cargo from premature degradation.

**Figure 1:**
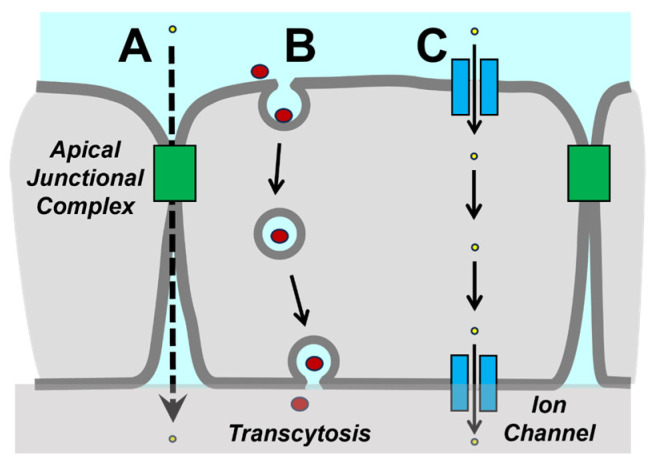
Pathways across epithelial barriers. Shown are the paracellular route (between cells) regulated by the apical junctional complex (A), transcytosis (B), and the transcellular route (through cells) mediated by ion channels (C).

In this review, we first discuss molecular components that reflect the current understanding of barrier function and describe two predominant routes of transit across the barrier: paracellular permeability and transcytosis (Figure 1). Technologies engineered for drug delivery are discussed, highlighting unique and shared elements related to targeting different tissues. Finally, we highlight future directions and opportunities for using nanostructured technologies to further optimize transepithelial therapeutic delivery.

## 2. Tight Junctions and Paracellular Diffusion

**Figure 2:**
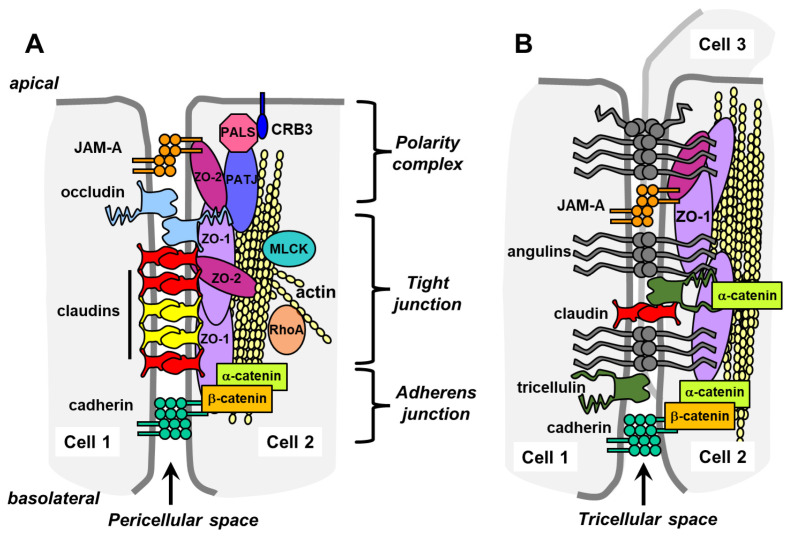
The apical junctional complex (AJC) and tricellular junctions. (**A**) Shown are major functional zones of the AJC, including the polarity complex, tight junctions, and adherens junctions. A common theme for the structure of the AJC are layers of transmembrane proteins complexed to scaffold proteins, such as zonula occludens (ZO)-1 and ZO-2, that crosslink them to cortical actin cytoskeletal filaments. Additional scaffold proteins are present in AJCs but, for simplicity, omitted from the diagram. Adapted from [14], used with permission. (**B**) Tricellular junctions consist of a combination of proteins found in bicellular junctions (such as claudins) and proteins unique to tricellular junctions, such as angulins and tricellular junctions.

Cell polarity is fundamental to epithelial cell function and results from preferential delivery of proteins to either the apical (top) or basolateral (bottom) surface of cells. Cell–cell contacts help define cell polarity and regulate paracellular permeability by a structure called the apical junctional complex (AJC). The AJC has several structural domains with discrete functions, including a protein complex that establishes cell apical/basal polarity, tight junctions that restrict movement of ions, large molecules, and pathogens through paracellular space, adherens junctions that regulate cell-to-cell adhesion and gap junctions that enable intercellular communication by the diffusion of small molecules and ions between adjacent cells (Figure 2) [6,7,8,9].

Tight junctions form at sites of cell-to-cell contact in the plasma membrane just below the apical surface, working to regulate the transport of ions, water, and soluble molecules between cells through the paracellular pathway sometimes referred to as tight junction “gate” function [10,11]. Tight junctions also have a distinct “fence” function, which regulates the diffusion of membrane lipids and transmembrane proteins between the apical and basolateral domains [12,13]. Tight junction gate and fence functions are independently regulated, and it has been demonstrated that fence function does not require tight junctions to have an intact gate function [12].

### 2.1. Transmembrane Tight Junction Proteins

There are two types of tight junction contacts, bicellular and tricellular tight junctions, which have unique structures [15,16]. Bicellular and tricellular junctions share some common features in that they are composed of transmembrane proteins tethered to cytosolic scaffolding proteins that are linked to the actin cytoskeleton [17,18]. However, tricellular junctions have unique features and protein composition that are distinct from bicellular junctions that influence their ability to regulate paracellular permeability.

**Figure 3:**
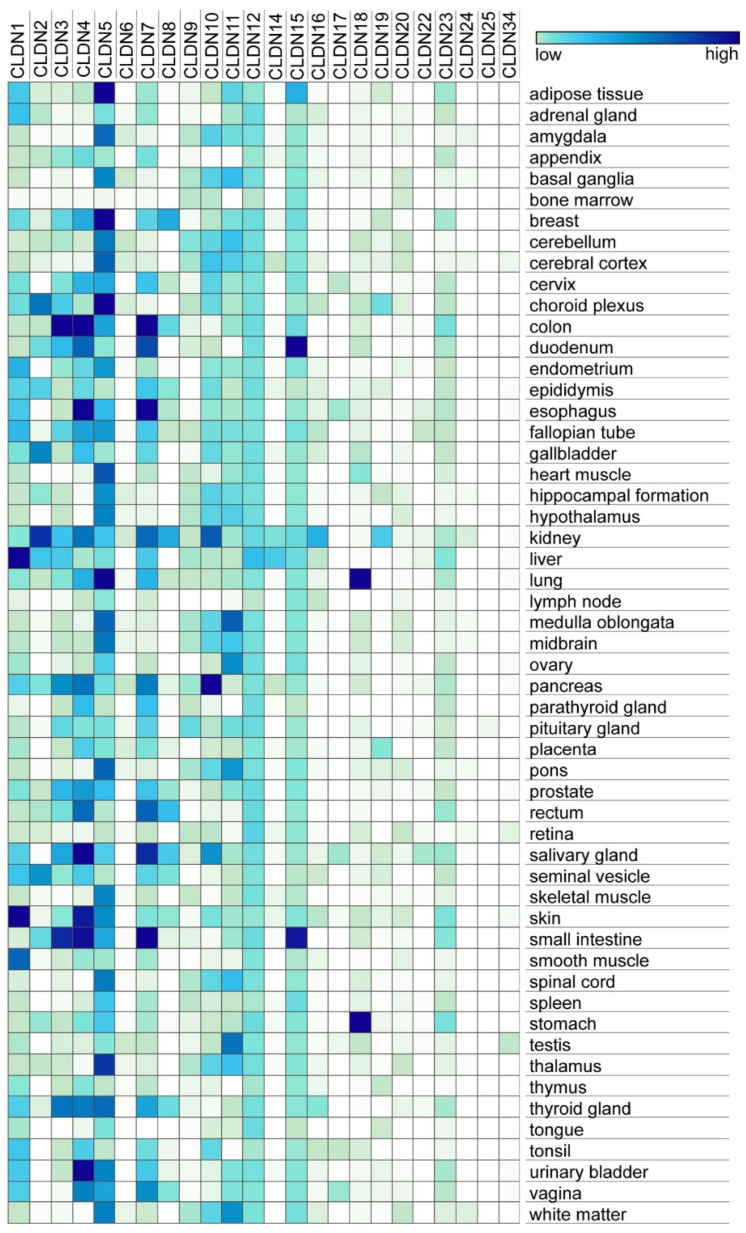
Expression of claudin genes by tissue type. RNA consensus data, which contains RNA transcript expression levels observed in 54 different human tissues from HPA and GTEx, were obtained from the Human Protein Atlas website. These data were previously normalized by HPA, and normalized gene expression values were calculated as the maximum nTPM value for each gene in the two data sources. The datasets were then screened and streamlined to obtain a list containing the normalized RNA expression levels for 24 claudin (CLDN) genes using Python ver 3.11. Matrix visualization platform by the Broad Institute, Morpheus, was used to visualize claudin gene expression by tissue type. From [28], used with permission.

The predominant family of proteins that regulate barrier permeability in bicellular tight junctions is claudins, which either provide a sealing component or form paracellular channels [19,20]. There are 27 mammalian claudins that are differentially expressed in a tissue-specific manner which accounts for differences in paracellular permeability (Figure 3). Nearly all claudins have a C-terminal PDZ binding motif that directly binds to zonula occludens (ZO) scaffolding proteins (ZO-1, ZO-2, and ZO-3). ZO-1 is the predominant scaffold protein crosslinking claudins to the actin cytoskeleton [21]. Claudins also interact with cytosolic proteins involved in cell signaling, which can influence cell behavior as well as paracellular permeability [22,23].

Claudins can be categorized functionally based on their apparent ability to form paracellular pores, thereby facilitating paracellular ion and water permeability, or by their ability to decrease paracellular permeability. The ion channels formed by claudins fall into several different categories, where they can be either anion- or cation-permeable, depending on the amino acid composition of their extracellular domains [19]. Some claudins also have a barrier-forming effect that can be anion or cation specific. In addition, claudin-2 and claudin-15 have also been demonstrated to form water channels [20,24]. Besides forming homomeric channels, claudins can also interact heterotypically (between cells) and heteromerically (within cells) [25]. Since a paracellular ion channel is formed by at least four claudins, the ability of claudins to intermix enables the formation of channels with unique permeability characteristics that are not attainable by channels formed by a single claudin [26,27].

When tight junctions are visualized by freeze-fracture electron microscopy or super-resolution fluorescence microscopy, claudins are organized into a series of strands that are interconnected by branchpoints, where the extent of branching depends on the amount of expression of occludin, a tight junction-associated MARVEL protein [29]. Tight junctions composed of sealing claudins that completely block paracellular ion diffusion show barrier function that increases with increasing tight junction strand count and branching. However, this is not the case when considering tight junctions containing claudins that form ion channels, where more stands could be associated with higher ion permeability [21,30].

The Ig superfamily transmembrane protein JAM-A serves several roles in regulating tight junction permeability. It facilitates tight junction formation by recruiting ZO-2 and, indirectly, ZO-1 to nascent junctions as well as other cofactors [31]. JAM-A also directly contributes to barrier function, specifically by impeding the paracellular diffusion of macromolecules 4 kDa or larger [32]. This contrasts with claudins, which can limit the diffusion of molecules that are smaller than 0.5 kDa, including water, small carbohydrates, and amino acids. Thus, any strategy to enhance the paracellular permeability of macromolecules across the bicellular route will need to target both claudins and JAM-A.

### 2.2. Tricellular Junctions

In contrast with bicellular tight junctions, tricellular tight junction structure and permeability are regulated primarily by angulins, single-pass transmembrane proteins which were initially discovered as lipolysis-stimulated lipoprotein receptor (LSR) family proteins [33,34]. Angulin-1 is the most widely expressed isoform and has been shown to interact with ZO-1 and other scaffold proteins to engage the cytoskeleton. Tricellular junctions also incorporate proteins such as claudins, although to a lesser extent than bicellular junctions (Figure 3) [35].

Tricellular junctions have been demonstrated to provide an interface that enables small molecule permeability but does not regulate ion permeability [16]. The relative role of the tricellular pathway in regulating paracellular permeability differs and is especially critical for paracellular water permeability in monolayers containing high-resistance bicellular tight junctions [34]. In addition to angulins, the MARVEL protein tricellulin is also highly localized to tricellular junctions. However, tricellulin is not required to regulate tricellular junction permeability and instead organizes tricellular tight junction strand architecture, a role similar to that of occludin for bicellular tight junction strands [29,35].

Tight junctions are instrumental in regulating the movement of small molecules, ions, and water in the paracellular space. When considering the design of a pharmacologic agent to target the paracellular route, tight junctions must be considered. However, movement across epithelial cell monolayers is controlled by the combination of paracellular permeability and the ability to move through cells via transcytosis.

## 3. Transcytosis

Although the paracellular route can accommodate the transport of molecules less than 4 kDa, larger molecules are not efficiently transported by the paracellular route, even when tight junctions are completely disassembled by calcium depletion [36]. Instead, large macromolecules are more effectively transported across epithelial barriers by transcytosis. Transcytosis is particularly important for immunoglobulin (Ig) transport [37], which is important physiologically as part of the adaptive immune response and pharmacologically as a pathway for the uptake of antibody-based biologics.

Of most importance to the transport of pharmacologic biologics are the so-called epithelial neonatal Fc receptors (FcRn), which bind to IgG at the apical surface, leading to substrate endocytosis and subsequently transcytosis and basolateral release [38,39,40]. Despite the name, FcRn is broadly expressed by adult epithelia and so can be an effective target for macromolecular therapeutics. In addition to IgG, FcRn can also bind albumin and mediate its transcytosis, suggesting a broad potential to design pharmacologic agents based on proteins beyond IgG [38,41].

In addition to FcRn, a low-affinity IgA receptor has also been found to be expressed by intestinal epithelial cells, which may mediate the apical to basolateral transcytosis of monomeric IgA [42]. This IgA receptor is distinct from the polymeric IgA/IgM receptor (pIgR), which mediates the basolateral to apical transport of dimeric IgA and IgM [43]. Although pIgR and FcRn differ in directionality, their trafficking in cells shows some overlap, particularly in the rab11a positive endosomes and other vesicles associated with the apical plasma membrane recycling pathway [44]. Apically delivered pIgR is largely restricted to early and recycling endosomes, which helps ensure the basolateral to apical directionality of ligands mediated by pIgR trafficking. The directionality of pIgR ligand delivery is also supported by the mechanism of ligand release by proteolytic pIgR cleavage that prevents subsequent binding of apical ligands [45]. These two mechanisms make it unlikely that pIgR ligands could be engineered that would traffic in an apical to basolateral direction. By contrast, FcRn has less stringent directionality. Instead, net transport is driven by higher levels of ligands in the apical vs. basolateral environments [44].

Consistent with a role in the immune response, several cytokines have been identified that upregulate the expression of pIgR, including interleukin-1 (IL-1), interleukin-17 (IL-17), interferon-γ (IFN-γ), and tumor necrosis factor-α (TNF-α) [37,45]. Proinflammatory stimuli have also been identified that upregulate FcRn expression, predominantly through NFkB signaling [46,47,48]. The ability to regulate receptor expression suggests the potential to improve transcytosis of biologics in inflammatory diseases or by application of agents that increase FcRn expression.

## 4. Transdermal Drug Delivery

Transdermal drug delivery is an increasingly popular method for drug delivery, with recent innovations leading to improved tissue permeability of a broader range of drugs, enhanced controlled release, and targeted delivery [49]. This approach provides an alternative to other routes of delivery, such as oral, intravenous, and intramuscular. Methods of transdermal delivery can be non-invasive, self-administered, decrease the required dosing frequency, and facilitate a steady plasma concentration of therapeutic in comparison to other routes. Additionally, transdermal delivery avoids first-pass metabolism by the liver, which can prematurely degrade drugs delivered orally [50]. However, the protective structure of the skin barrier can make drug delivery challenging.

**Figure 4:**
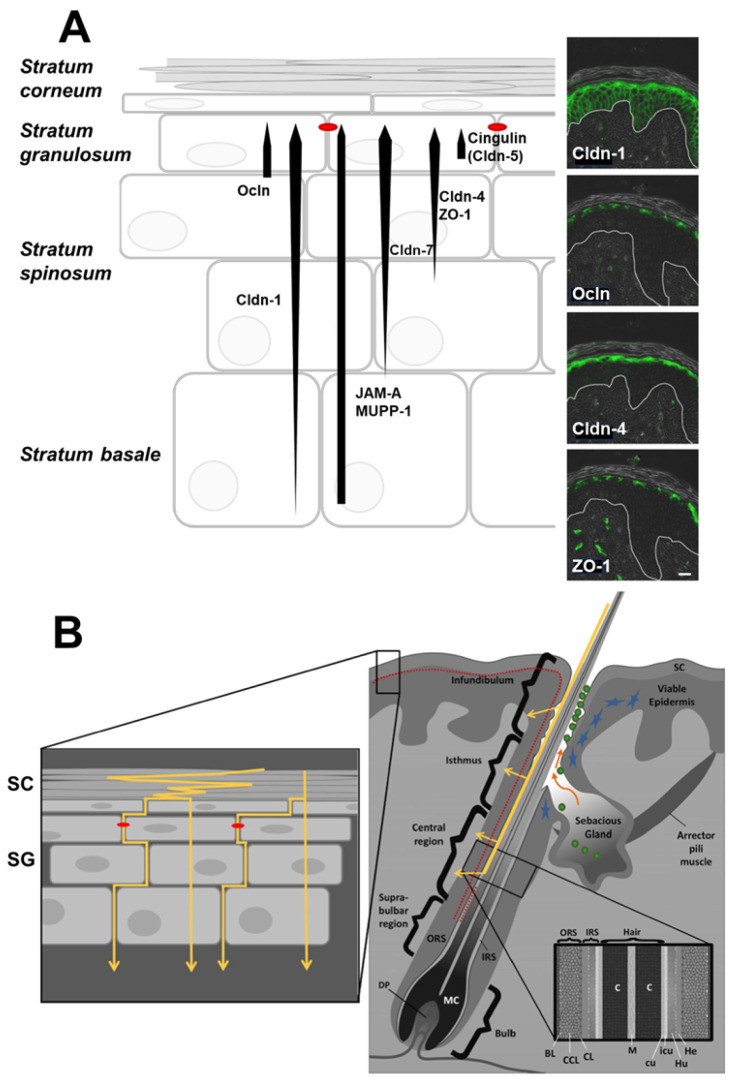
Schematic overview of the mammalian epidermis. (**A**) Tight junction (TJ) protein localization pattern and immunohistochemical staining (green) of claudin-1 (Cldn-1), occludin (Ocln), claudin-4 (Cldn-4), and zonula occludens protein-1 (ZO-1) (overlay of immunofluorescence staining and phase contrast pictures). Red dots denote functional TJ structures. JAM-A, junctional adhesion molecule-A, MUPP-1, multi-PDZ domain protein 1. Bar 20 μm from [59], used with permission. (**B**) Putative penetration pathways of the skin. BL: basal cell layer of HF, C: cortex, CCL: central cell layer of HF, CL: companion cell layer of HF, cu: cuticle of hair shaft, He: Henle’s layer, Hu: Huxley’s layer, icu: cuticle of IRS, IRS: inner root sheath, M: medulla, MC: matrix cells, ORS: outer root sheath, SC: stratum corneum, and SG: stratum granulosum. Yellow arrows: putative paracellular and transcellular penetration pathways. Red dots: tight junctions, blue stars: Langerhans cells, orange arrows: sebum, and green circles: microbiota. From [56], used with permission.

The outermost surface of the epidermis consists of a keratinized stratified squamous epithelium that provides an effective barrier to the external environment. The most outward-facing layer of skin cells is the stratum corneum, a cornified layer of compressed cells enriched for waxy barrier-forming proteins, including loricrin, involucrin, and filaggrins [51]. Tight junctions are restricted to the stratum granulosum, which is a layer of squamous cells just beneath the stratum corneum [52] (Figure 4). Although some tight junction proteins, such as occludin, are restricted to epidermal tight junctions, others, including claudin-1 and claudin-4, are present in other layers, suggesting a functional role in skin homeostasis beyond barrier formation [53]. Claudin-1 is essential for skin barrier function, as evidenced by claudin-1 null mice, which die of dehydration soon after birth and claudin-1 mutations associated with the human disease neonatal ichthyosis-sclerosing cholangitis (NISCH) syndrome [54,55]. Decreases in claudin-1 and epidermal barrier function are also observed in skin disease states such as atopic dermatitis and psoriasis, which are pathological yet also have the potential to promote transdermal drug delivery [56,57].

In addition to primary routes of paracellular transit and transcytosis through the dermal layers, which are considered in other epithelial tissues, the appendageal route is an additional consideration in the skin. This route consists of hair follicles, sebaceous glands, and sweat glands as routes of delivery, which can serve as ducts through the upper layers of the skin. Characterization of porcine hair follicles [58] and human hair follicles [57] suggests that continuous functional barrier tissue, regulated by tight junctions, lines the outer root sheath of hair follicles. The upper region of hair follicles possesses two barriers, tight junctions, and the stratum corneum, whereas, in lower areas of the hair follicle, tight junctions serve as the only barrier [57,58]. While hair follicles represent a limited portion of the skin surface area, follicle-based delivery is an interesting consideration for drug delivery due to the possibility of bypassing the stratum corneum.

### 4.1. Topical Agents and Microneedles

Both the external and tight junction barriers must be breached in order to have effective transdermal drug delivery [60]. Unassisted topical delivery is generally limited to lipophilic therapeutic molecules that are small, less than a few hundred Dalton, and require low drug mass to achieve therapeutic effect [50]. In addition to subdural bolus injection, methods to introduce pharmacological agents past the skin barrier include electroporation, microneedles, chemical permeation enhancers, fractional laser ablation, and sonophoresis [61,62]. A drawback of several of these active delivery methods is reliance on clinical equipment, which limits patient self-administration [63]. Injury to the stratum corneum can also provide a route for drug application, particularly for treatments that promote wound repair [56,57].

Although unmodified silicon microneedles can readily penetrate the skin, they do not provide significant enhancement of drug delivery beyond bolus injection. However, coating microneedles with a specific nanostructured surface pattern using polyether ethyl ketone (PEEK) has been shown to enhance drug delivery across the dermal layer and entry into the vascular and lymphatic circulation [64,65,66]. Nanostructured PEEK stimulates a breakdown of dermal tight junctions via an integrin-dependent pathway that stimulates rearrangements of the actin cytoskeleton via activation of myosin light chain kinase [66,67]. How nanostructured PEEK stimulates delivery into the circulation is as yet unknown, but may involve stimulation of transcytosis across the vascular surface [36]. Other microneedle platforms use engineered nanopores, stimuli-responsive polymers, or contain secondary delivery technology, such as microneedle-loaded nanoparticles, as a means to facilitate controlled drug release after breaching the initial layers of the skin [68].

### 4.2. Nanohydrogels

Nanohydrogels are another vehicle developed to facilitate the delivery of pharmacologically active agents that can cross the skin barrier [69,70]. Nanogels are crosslinked, nanoscale, three-dimensional hydrophilic polymeric networks [69]. The polymeric materials that nanohydrogels are composed of are selected for being non-adhesive, biocompatible, and biodegradable. Nanohydrogels possess several advantageous qualities, such as their hydrophilicity, flexible design allowing versatile loading, and prolonged holding capacity [71,72]. Nanohydrogels can be designed to possess stimuli-responsive elements, such as thermoresponsive or pH-responsive polymers, to facilitate controlled drug release based on the environment of a target layer of the skin or of a hair follicle [73].

In order to accommodate a variety of drugs while maintaining biocompatibility and controlled release, nanohydrogel composition can be adjusted to facilitate different methods of encapsulation or conjugation. For instance, baicalin is a medicinal plant derivative widely used in East Asian countries for the treatment of various inflammatory diseases. Manconi et al. [74] found that nanohydrogels made of gellancholesterol are able to be loaded with baicalin, resulting in a significant effect on wound healing. This is consistent with recent studies that have reported the low bioavailability of baicalin, especially when applied topically, providing a rationale for the use of hydrogels as a topical delivery system for baicalin [74,75].

### 4.3. Inorganic Nanoparticles

Many properties of inorganic nanoparticles have been studied for transdermal delivery. Inorganic nanoparticles include nanoparticles fabricated of silica, metals, metal oxides, quantum dots, and more. In many cases, inorganic nanoparticles can be used not only as therapeutic nanocarriers but also as diagnostic tools and photothermal transduction agents [76]. Gold nanoparticles (Au-NP) have favorable surface chemistry for functionalization, can be produced across a range of sizes, and demonstrate good biocompatibility and low toxicity [77]. Transdermal penetration of gold nanoparticles has been identified to be size dependent, with several studies identifying their smallest particle investigated demonstrating greater permeation than that of larger particles [78,79].

Charge and surface modification have also been identified to be an influential factor but with mixed conclusions across studies [77]. For example, Hao et al. examined the effect of Au-NP charge on the mechanism of transepithelial permeation, comparing positive, negative, and neutrally charged Au-NP. Their results suggest that positively charged Au-NP penetrates tissue more efficiently and transit via a combination of paracellular leak and transcytosis [80]. In contrast, Chen et al. found Au-NP functionalized to be negatively charged to reach the greatest penetration for topical delivery of vascular endothelial growth factor (VEGF) via Au-NP [81]. Other studies also indicate the significance of intercellular transit of gold nanoparticles [82]. Tak et al. examined the shape dependence of silver nanoparticle skin penetration. Interestingly, their results located penetration of rod-shaped, spherical, and triangular AgNPs to capillaries in vivo, while in vitro results identified shape dependence, with rod-shaped reaching the dermal layer, followed by spheres reaching the epidermal layer. Transmission electron microscopy (TEM) results suggested a paracellular pathway of penetration by AgNPs [83].

### 4.4. Chitosan-Coated Nanoparticles

Nanoparticles can also be incorporated into composite systems to promote drug delivery [84]. As an example, platforms using mesoporous silica nanoparticles for delivery have been paired with chemical permeation enhancers, such as deep eutectic solvents, to temporarily disrupt skin structure [85]. Alternatively, mesoporous silica nanoparticles have been incorporated within composite systems, such as loaded within a carboxyl chitosan and oxidized pullulan gel to allow for skin penetration for the treatment of osteoarthritis. In this system, the chitosan-based gel allowed for the transdermal delivery of colchicine-loaded mesoporous silica nanoparticles (MSNs) for the treatment of osteoarthritis [86].

Both natural and synthetic polymeric nanoparticles have been designed for transdermal delivery. Chitosan, a natural polymer derived from chitin, has been used extensively for dermal drug delivery applications, enhancing drug delivery via several mechanisms. Exposure to chitosan can change the conformation of keratins, a component of the keratinocyte cytoskeleton, reducing stratum corneum barrier cohesion. Treatment with chitosan also induces changes to intercellular lipids, altering the structure of the stratum corneum to increase permeability [87]. Modified chitosan materials can be used to increase skin hydration, which also increases transdermal permeability. In intestinal epithelial tissues, treatment of chitosan has been found to induce reversible tight junction opening and the redistribution of claudin proteins. These findings may translate to tight junctions in the granular layer of the skin [87].

Recently, Wenjun Zhu and colleagues successfully delivered multiple classes of biomacromolecules transdermally using a fluorocarbon-modified chitosan (FCS) nanocomplex platform [88]. In this work, fluorocarbon-modified chitosan was complexed to either antibodies or antigens. The authors suggest that both classes of nanocomplexes reach the dermis of the skin via the transcellular and transappendageal routes. When treated with FCS nanocomplexes, model monolayers of human skin epidermis cells (HACAT) demonstrated a drop in TER, which recovered to its original barrier function after 12 h, temporarily opening the paracellular route. Transmission electron microscopy (TEM) imaging of skin demonstrated opening of tight junctions as well as enlargement of intracellular spaces. Discontinuity of ZO-1 was observed, as well as phosphorylation of the myosin light chain. FCS nanocomplexes were observed to colocalize with keratin 14, indicating localization in hair follicles and sweat glands. The group reported that the nanocomplexes containing fluorocarbon chains that are neither purely hydrophobic nor hydrophilic create a complex that is less sticky when penetrating barriers. When loaded with anti-PDL1 for the treatment of melanoma, delivery via FCS nanocomplexes demonstrated the slowest tumor growth and longest survival in a mouse tumor model when compared to injected anti-PDL1. The group also demonstrated the versatility of their platform by demonstrating elevated SARS-CoV-2 vaccine titers in both the FCS nanocomplex delivery and injection of SARS-CoV-2 vaccine in mice [88].

### 4.5. Lipid-Based Nanoparticles

Lipid vesicular carriers have been extensively studied for topical delivery applications. Liposomes are deformable phospholipid bilayer spheres enclosing an aqueous center. Due to the amphiphilic character of phospholipids, liposomes are conducive to the delivery of both hydrophilic and lipophilic therapeutics. Most unmodified liposomes do not penetrate the skin beyond the upper layer of the stratum corneum. Liposomes ≤70 nm have been observed to reach the epidermis and the dermis [89]. Penetration is improved by chemical or mechanical modifications to the lipid bilayer structure. These modified systems include transfersomes, which incorporate an edge activator (typically a surfactant), ethosomes (which incorporate ethanol), and niosomes, which are bilayers of nonionic surfactants. Niosomes are able to loosen and increase the permeability of the stratum corneum to improve delivery [90].

Lipid nanoparticle systems, solid lipid nanoparticles (SLNs), and nanostructured lipid carriers (NLCs) present many advantages for therapeutic delivery, as they are very stable, biocompatible, biodegradable, facilitate extended drug release, as well as are advantageous to manufacturing. SLNs are particles composed of solid lipophilic matrix at room temperature, which is stabilized with surfactant, and can be used to encapsulate lipophilic or hydrophilic drugs but demonstrate limited loading capacity and can release drug while being stored. NLCs improve upon the limitations of SLNs, increasing loading capacity and stability. NLCs enclose a liquid phase within a solid phase [90,91]. SLNs and NLCs enhance skin permeation of therapeutic by creating a high concentration of therapeutic at the surface of the skin due to the surface area of contact, forming an occlusive film at the surface of the skin, maintaining skin hydration, which increases permeation [92].

## 5. Ocular Drug Delivery

**Figure 5:**
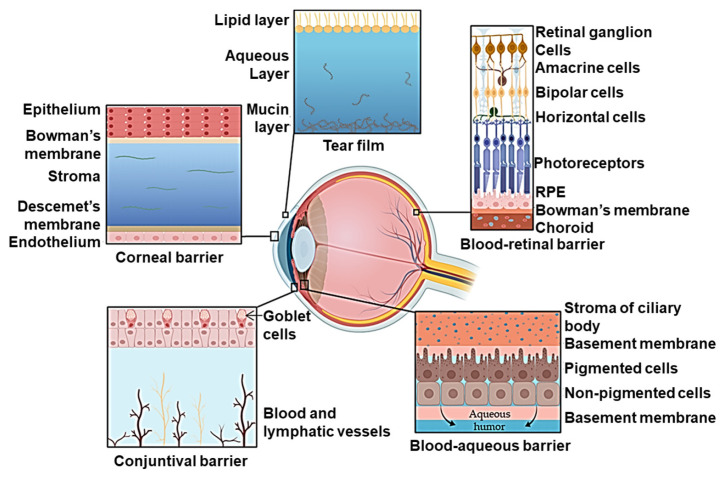
Barriers to ocular drug penetration. The tear film is composed of three layers: the lipid layer, the aqueous layer, and the mucin layer. The corneal layer is formed by epithelium, Bowman’s membrane, stroma, Descemet’s membrane, and endothelium. The conjunctival barrier is vascularized. The blood–aqueous barrier starts on the stroma of the ciliary body and is composed of the basement membrane, pigmented cells, and nonpigmented cells and delimited by the basement membrane. The blood–retinal barrier is formed by retinal ganglion cells, amacrine cells, bipolar cells, horizontal cells, both types of photoreceptors, retinal pigment epithelium (RPE), and the Bowman’s membrane. From [98] under the terms and conditions of a Creative Commons Attribution (CC BY) license (https://creativecommons.org/licenses/by/4.0/ accessed on 25 March 2024).

The corneal epithelium forms a barrier that protects the eye from chemical, biological, and physical insults from the environment. Located at the front of the eye, the cornea protects other structures in the eye from foreign substances [93] (Figure 5). The paracellular barrier in the corneal epithelium is primarily provided by tight junctions [94], while the transcellular pathway is controlled by epithelial components such as the mucin layer and glycocalyx [95]. Tight junctions in the eye are highly dynamic and inflammatory signaling has been found to be responsible for alterations of the corneal epithelial barrier [96]. Knowledge in understanding tight junctions and ocular epithelial barrier function may provide insights into therapeutic strategies to treat ocular barrier diseases such as allergies, infectious keratitis, and dry eye disease [97].

Visual impairment and eye conditions leading to the loss of sight, such as macular degeneration and glaucoma, exert a profound negative impact on a patient’s quality of life [99]. The last several decades of research have yielded substantive gains in understanding ocular diseases and the development of treatments, many of which are macromolecular therapeutics [100,101].

Several physiological barriers hinder therapeutic access to the intravitreal space, the target tissue for most ocular therapeutics. Drug delivery into the inner eye is restricted by tissue and fluid eye barriers [102]. Current therapies rely on delivery via periocular injection, intraocular injection, and topical application [103]. For the topical delivery systems, the first barrier is the tear film, at which point the applied therapeutic is subject to flow as the tear film turns over, draining systemically prior to entering the target ocular tissue [101]. Therapeutics then must traverse the cornea, the most significant mechanical and chemical barrier to ophthalmic drugs. The cornea is composed of three layers: the outermost layer is the corneal epithelium, a barrier maintained by tight junctions, followed by the stroma and endothelium [100,101]. Bioavailability is further challenged by the expression of efflux transporters in the corneal epithelium. These barriers motivate the bypassing of these barriers by delivery via intravitreal injection. Systemic delivery of ocular-targeted therapeutics is met with the blood–retinal barrier, composed of the retinal pigment epithelium and the retinal capillary endothelial layer in the posterior region of the eye. Despite advances in ophthalmic drug formations, the delivery of medication is limited by diffusion across the corneal barrier.

In addition, properties of corneal barrier tissue limit the types of therapeutics that are able to transit the barrier via diffusion. Paracellular pores in the corneal epithelium have been modeled to be approximately 1.6 nm in diameter, which restricts diffusion across tight junctions to molecules less than 500 Da [104]. The lipophilic character of the epithelium favors the passage of lipophilic molecules, while the underlying stroma layer, composed of collagen fiber bundle sheets, favors the passage of hydrophilic molecules [105,106]. The negatively charged mucin layer that covers the corneal epithelium favors the transit of cationic materials [105]. The combination of these properties greatly restricts the therapies that can be topically applied and reach meaningful intraocular concentrations.

### 5.1. Topical Solutions and Implant-Based Drug Delivery

Due to physical barriers intrinsic to ocular anatomy, delivery of many therapeutics to the eye is limited to eye drops applied to the surface of the eye and intravitreal injections to the anterior region of the eye [100,101,107]. Eye drops are a highly inefficient delivery system, with only 0.07–4.3% of small molecule therapeutics reaching the anterior segment of the eye and even less reaching the posterior segment [101,108,109]. These drops must also be administered frequently, challenging patient compliance. Alternatively, intravitreal injections are highly invasive, presenting an opportunity for the introduction of infection to the eye or ocular hemorrhage. Intravitreal injections must be performed in-clinic and are understandably uncomfortable for the patient, also challenging patient compliance.

Surgically implanted refillable drug reservoir devices for sustained ocular delivery are an emerging alternative to intraocular injections, with Genentech’s Port Delivery System concluding phase 3 trials, but this approach remains invasive [103]. Alternatively, engineered nanoporosity allows for sustained or controlled release of therapeutics from biodegradable implantable devices to serve as an alternative to intravitreal injection [110,111]. Ocular delivery presents a high-impact opportunity for nanostructure-mediated delivery to enable improved therapeutic delivery.

### 5.2. Optimizing Nanocarriers for Ocular Drug Delivery

The design of nanoscale carriers can facilitate the passage of therapeutics that would not ordinarily pass the aforementioned barriers. Here, the discussion of particle-based systems will be limited to those interacting with the epithelium. Nanostructure-mediated approaches to improve ocular delivery of biologics include nanoparticle systems and nanotopography for enhanced adhesion and transepithelial penetration.

Many nanomaterials have been developed as a means to extend the residence time of therapeutics. These include topical nanocarriers, which serve to extend topical drug exposure/precorneal retention of therapeutic despite tear film turnover. Many of these strategies leverage controlled release properties of nanocarriers or employ properties that enhance corneal affinity or allow for mucoadhesion to extend residence time. Nanocarrier design strategies for increasing the affinity of nanoscale materials to the surface of the cornea include using amphiphilic materials to interact with the lipophilic surface of the epithelial surface of the eye or using cationic materials to interact with the anionic cellular membrane [112]. Platforms used for these topical strategies include nanowafers, gels and nanogels, and mucoadhesive controlled release particle systems. These methods to increase precorneal retention of drugs rely on diffusion and are best suited for use with small molecule therapeutics. Alternatively, nanomaterials are also used in the intravitreal space, deposited via injection, to extend the efficacy of therapeutics via controlled release methods in order to reduce dose frequency and maintain concentrations of therapeutics.

### 5.3. Charged and Coated Nanomicelles

Because the ocular mucin layer is negatively charged, positively charged materials can be used to transiently reduce barrier function by opening tight junctions. Alternatively, “super-cationic” materials have been used to encourage transcorneal transcytosis [112,113]. Nanomicelles are composed of amphiphilic molecules that self-organize in an aqueous solution to form organized supramolecular structures. Micelle structures form by hydrophobic segments joining to make a core region, with a hydrophilic segment forming a shell interacting with water molecules. Micelle structures can be adapted for delivery requirements. Paired with polymer technology described above, nanomicelles can serve to deliver medication to the anterior segment of the eye [102].

Drug release from polymeric micelles follows two main mechanisms: (i) dissociation followed by drug cleavage or (ii) drug cleavage inside the micelle followed by diffusion out of the carrier. Lower rates of dissociation and diffusion can also be achieved via crosslinking of the micelle and the use of bonds between core-forming blocks and the drugs. Most polymeric micelles are coated with hydrophilic poly(ethylene glycol) (PEG) to form the shell of the micelle. The hydrophobic component usually consists of amphiphilic di-block (hydrophilic–hydrophobic) polymers, triblock (hydrophilic–hydrophobic–hydrophilic) polymers, graft (hydrophilic–hydrophobic), and ionic (hydrophilic–ionic) copolymers [114].

Using nanomicelles coated with a permeation-enhancing polymer, such as PEG or chitosan, allows for tight junction opening in the corneal epithelium, allowing for drugs to reach the anterior segment. Pepić et al. demonstrated that a chitosan-coated nanomicelles decreases transepithelial electrical resistance (TER) of human colon cell line (Caco-2) monolayers more than the nanomicelle on its own, indicating an enhanced decrease in tight junction integrity [115], suggesting a comparable mechanism for delivery across ocular tight junctions. Nanomicelles have also been developed to reach the posterior segment of the eye following topical application.

Recently, chitosan oligosaccharide-valylvaline-stearic acid-based nanomicelles were evaluated for the ocular delivery of dexamethasone after topical application. The particles were designed to actively target peptide transporter-1 to enhance permeation and were found to primarily traverse the conjunctival route. Clinically relevant concentrations of dexamethasone were identified to have accumulated in the posterior region of the eye, specifically the sclera–choroid–retina [116].

In another study, synthetic nanomicelles, fabricated using copolymer composed of PEG, poly(propylene glycol), and poly(ɛ-caprolactone) segments, loaded with aflibercept, an anti-VEGF therapeutic, were found to be capable of delivering clinically relevant concentrations of aflibercept to the retina following topical application. TER and the localization of ZO-1 were undisrupted by particle transit, suggesting that the nanomicelles may have traversed the cornea via transcytosis [117].

### 5.4. Polymeric Nanoparticles

Polymeric nanoparticles can be produced using natural or synthetic polymers and via a variety of fabrication methods to produce particles of varied characteristics. These design choices can be used to enhance ocular epithelial delivery. Particles can be designed with passage via the paracellular or transcytosis routes in mind; alternatively, particles can be targeted to the epithelial tissue directly to treat the ocular surface. For example, Contreras-Ruiz et al. designed cationic gelatin-based nanoparticles to deliver plasmids coding for a modified MUC5AC protein, a glycoprotein implicated in dry eye disease. Delivery and transfection via the particles increased expression of the glycoprotein and led to disease improvement in a mouse model. Specifically, the mouse corneal epithelial integrity was restored, with alterations observed in ZO-1, ZO-2, and actin [118].

Recently, the research group of Zhuang Liu and colleagues has developed several zwitterionic polymer–drug nanocomplex platforms to overcome ocular epithelial barriers [103,119]. In the first platform, Shen et al. [119] formulated nanocomplexes of FCS self-assembled with protein therapeutics. FCS nanocomplexes were identified to modulate barrier properties of the model corneal barriers as well as demonstrate therapeutic efficacy against two conditions. FCS nanocomplexes delivering anti-VEGFA were demonstrated to inhibit vascular proliferation and delivering anti-PDL1 to demonstrate antitumor immune response against choroidal melanoma. In the second platform, Jiang and colleagues used nanocomplexes of zwitterion-grafted chitosan (CS-ZW) as a delivery vehicle within eyedrops for therapeutic proteins for dry age-related macular degeneration (dAMD) [103]. They also demonstrated that the application of CS-ZW-nanocomplexes reduced TER by 30–40% and decreased the morphologic continuity of ZO-1 in Transwell barrier models composed of human corneal epithelial cells and human conjunctival epithelial cells. These barrier-altering effects were observed to be reversed following the removal of the CS-ZW nanocomplexes. These results suggest the CS-ZW nanocomplexes induced transient opening of the tight junctions, allowing for successful paracellular delivery of large molecular weight therapeutics to the retina. These results were reinforced in vivo, where Jiang et al. observed improvement in dAMD disease markers via qPCR and immunofluorescence staining of the fundus or back of the eye [103].

### 5.5. Inorganic Nanoparticles

Inorganic nanoparticles have been leveraged for transepithelial delivery in the eye. As a recent example, Luo and colleagues functionalized hollow ceria nanoparticles with chitosan for the purpose of opening tight junctions and with ZM241385, an adenosine receptor agonist that targets nanoparticles to the tissue of the ciliary body. In this case, the ceria nanoparticles not only serve as drug delivery vehicles but also serve a therapeutic purpose due to the antioxidant and anti-inflammatory properties of the ceria. Material interaction with tight junction was assessed via fluorescence microscopy, in which discontinuity of ZO-1 was observed following treatment with a nanoparticle system. The use of this platform resulted in 7 days of reduced intraocular pressure from treatment in comparison to commercially available eyedrops, which require multiple doses per day [120].

### 5.6. Lipid-Based Nanoparticles

Lipid-based nanocarriers have also been designed to create topical formulations capable of crossing ocular barrier tissues. Polyamidoamine (PAMAM)-coated liposome drug carriers have demonstrated permeability across the corneal epithelium and yielded a therapeutic response in the posterior chamber [121]. Recently, Qiu and colleagues designed a dendrimer-decorated, drug-loaded liposome in order to deliver latanoprost and timolol malate for the treatment of glaucoma. Utilization of amino-terminated PAMAM dendrimers imparts a positive surface charge and allows for enhanced mucoadhesion via polymer–mucin entanglements. In addition to extended precorneal retention, the group identified their positively charged liposomes demonstrated transit via the transcellular and paracellular routes. Immunofluorescence microscopy indicated that there was a reduction and discontinuity of ZO-1 in the positive liposome group in comparison to negatively charged and nontreatment controls. Single-dose administration of the liposomal carrier led to a 5-day reduction in intraocular pressure in rats, in comparison to an 8 h reduction following standard eye drop treatment containing free drug [122].

While we primarily focused on topically delivered nanoformulations and, therefore, epithelial barriers in the anterior region of the eye, engineered nanoscale systems have also been leveraged to access the eye across other epithelial barriers. A recent example is work by Bohley et al. [123], using lipid nanocapsules, mimicking the density of very low-density lipoprotein particles, which are naturally capable of traversing biological barriers. The nanocapsules were decorated with cyclo(-Arg-Gly-Asp-D-Phe-Cys) (cRGD) in order to facilitate passage across the choroidal endothelial barrier as well as the retinal pigment epithelial barrier. The group identified that cRGD was necessary for their nanocapsules to accumulate in the retinal pigment epithelium. The group also demonstrated that a single IV delivery of cyclosporin A via their cRGD lipid nanocapsules resulted in normal retinal development in a mouse model of retinopathy of prematurity [123].

## 6. Pulmonary Drug Delivery

**Figure 6:**
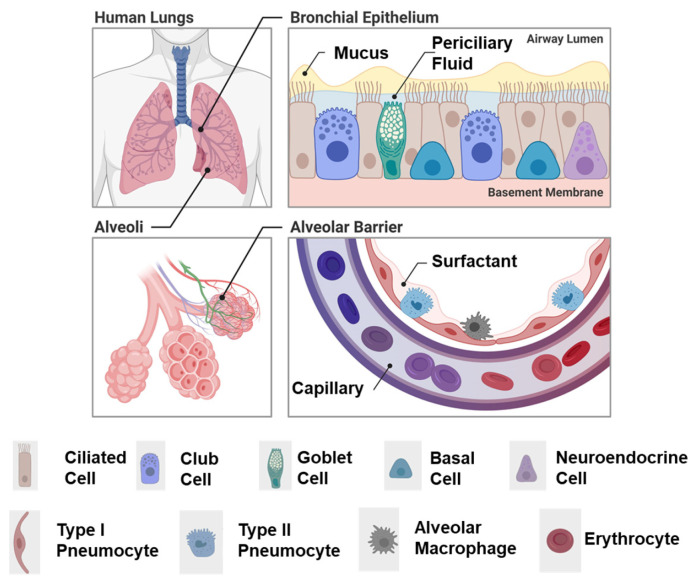
Epithelial barriers of the human respiratory tract. Cell composition of epithelial linings in the lungs varies in different segments. The pseudostratified columnar epithelium in the bronchial and bronchiolar region is composed of ciliated, club, goblet, basal, and neuroendocrine cells. The cell layer is covered by a thin layer of periciliary fluid and mucus. The alveolar epithelium is squamous in nature and comprises predominantly the extremely thin (for efficient gas exchange) AT1 and the cuboidal AT2 cells (responsible for production, secretion, and recycling of surfactant-proteins). Alveolar macrophages are also present. From [126] under the terms and conditions of the Creative Commons Attribution (CC BY) license (https://creativecommons.org/licenses/by/4.0/ accessed 25 March 2024).

The lung is a branched organ that can be divided into two major airway zones, the conducting airways and lower or alveolar airways, where gas exchange occurs [124]. The epithelium facing airspaces is heterogeneous and controls lung homeostasis by acting as a physical barrier, regulating fluid balance, metabolism, and tissue immunity [125,126] (Figure 6). Conducting airways consist of a branched network, often termed the “bronchial tree”, that is predominantly maintained by a system of ciliated and mucus-producing epithelial cells. The largest airway of the lung is the trachea, which begins at the upper neck and then passes through the upper body, where it branches into two main bronchi, followed by further branching into smaller and smaller airways, ultimately forming bronchioles. The trachea and bronchi are stabilized by cartilaginous rings; however, bronchioles are stabilized by smooth muscle. Bronchioles terminate at distal gas-exchanging sacs called alveoli (Figure 6). By contrast with the conducting airways, which are cleared by mucociliary function, alveoli are coated with an amphipathic fluid, a pulmonary surfactant, which maintains lower airspace integrity against the air–liquid interface needed for gas exchange [127].

For local pulmonary delivery, a therapeutic formulation must be paired with a device to facilitate inhalation. Inhaled medications must make their way through the entire bronchial tree and pass through numerous branches where they could potentially be deposited [128]. Another complication to inhaled delivery systems is that the respiratory tract has evolved protective mechanisms to keep inhaled particles out of the lungs and to remove or inactivate them once deposited [129]. Current pulmonary medicine utilizes inhaled drugs through a pressurized metered dose inhaler (pMDI) [130], dry powder inhaler (DPI), or forms of nebulizers [131,132]. Nanoscale formulations are constrained by the parameters of materials supported by current delivery devices and consideration of the aerodynamic radii compatible with lung airflow [132].

The innate defense mechanisms in the lung can be a barrier to drug delivery. Mucociliary clearance can remove drugs from the lungs before they are absorbed through the airway epithelium [133]. Lung mucus in the healthy lung clears deposited material within 24 h and can move medication from its target site, which can increase required dosing frequency [134,135]. Conversely, airway stenosis, or narrowing, due to various disease states can limit the extent of drug delivery and prevent deposition into target regions either by reducing airflow or by causing obstructions such as mucus plugging [136]. Proteolytic enzymes also may hydrolyze medications and inactivate drug particles [137].

Despite delivery obstacles, pulmonary epithelial delivery presents several advantages to systemic delivery. If the lung is the treatment target, therapy can be directed to tissue, reducing off-target effects and systemic side effect profiles. For treatment aimed at reaching the systemic circulation, the surface area of alveoli is large, approximately 100 m^2^, presenting a large tissue area for absorption, which is highly vascularized and has a relatively thin epithelial barrier, a thickness of only 0.1–0.4 μm [127]. While enzymatic degradation is still an obstacle, enzymatic activity is lower in pulmonary routes than in oral routes of delivery. Additionally, pulmonary delivery of therapeutics avoids the effects of first-pass metabolism on the therapeutic in comparison to the oral route. Aside from the tissue-level benefits, the use of inhaled therapies is non-invasive, and current delivery devices facilitate patient self-administration [135,137,138]. Nanomaterials are being studied as a means to circumvent the above-mentioned hurdles to pulmonary drug delivery.

### 6.1. Nanoscale Materials for Pulmonary Delivery

Nanomaterials are effective tools for the treatment of pulmonary disease. Developed pulmonary drug delivery platforms include liposomes, polymeric and inorganic nanoparticles, dendritic particles, nanogels, and nanocrystals [139]. Of these systems, liposomes, polymeric, and inorganic nanoparticles have been developed for the treatment of lung disease with liposomal and polymeric nanoparticles as the most useful for transepithelial pulmonary delivery [140]. Liposome-based drugs provide an extended therapeutic response and have the ability to incorporate both water-soluble and lipid-soluble molecules. The synthetic polymers PLGA and PLA are commonly used due to their biocompatibility, as well as the natural polymers chitosan and alginate [141].

Several of the aforementioned obstacles to pulmonary drug delivery can be accommodated through nanomaterial design. Nanomaterials are smaller than the 10 μm threshold at which coughing is induced. Size also influences the depth of deposition in the lung. Findings suggest diameters of 1–100 nm deposit in the alveolar region, whereas larger particles tend to be retained in the upper respiratory tract.

Macrophages in the alveolar epithelium make no distinction between harmful and beneficial particles in the lung and thus may clear agents before they have a chance to have a therapeutic effect [129,142]. Alveolar macrophages primarily phagocytose particles in the 1 to 5 μm range while not recognizing particles <200 nm [143]. However, negatively charged particles tend to avoid phagocytosis, whereas positively charged particles tend to bind to negatively charged acids on the surface of macrophages [144]. Surface modifications can also be used to reduce macrophage uptake, such as PEGylation, decoration with “self” peptides, or coating with cell membrane proteins [145].

Mucocilliary clearance can be accommodated via mucoadhesive or mucopenetrative design [135]. Mucopenetrative polymers include PEG, polydopamine (PDA), poly(vynyl alcohol), zwitterionic materials, and dextran, among many others [146]. Additionally, the restriction of particle size to the diameter of 100–200 nm allows for penetration through respiratory mucus networks [147].

Inhaled nanomaterial strategies show a great deal of promise, but the design must include a complete assessment of biosafety and assessment of degradation or clearance. Evidence suggests that prolonged accumulation of some nanoparticles can lead to the development of lung nodules or tumors. Toxicology studies assessing nanoparticles that are products of industrial pollution demonstrate that they have the capacity to damage lung epithelial cells or interact with essential pulmonary surfactant proteins [148].

### 6.2. Mesoporous Silica and Calcium Phosphate Nanoparticles

Mesoporous silica nanoparticles (MSNs) are becoming widely used as a drug delivery system for the lungs. The driving factor behind the use of MSNs rather than a liposome or polymeric nanoparticle is the ability to be functionalized with “molecular gates” or nanovalves that allow for the drug inside to be delivered in response to an external stimulus such as pH or receptor binding [140]. This allows for the controlled release of medication rather than diffusion or carrier decomposition, as seen for many liposomes and polymeric nanoparticles [140]. For instance, a functional aerosol to use for respiratory disease acute lung injury (ALI) and acute respiratory distress syndrome (ARDS) was developed using MSNs that were coated with polyethyleneimine (PEI) and functionalized with PEG. These aerosolized MSNs have been found to reach targets in the distal lungs and alleee inflammatory responses [149]. While conducted considering the context of silica nanoparticles as part of air pollution, recent work by Detampel et al. suggests that the transcytosis of 10–20 nm silica nanoparticles across alveolar epithelial barriers occurs via caveolin-initiated, myosin-dependent macropinocytosis [150]. MSNs are biodegradable. Following drug delivery, MSNs are degraded in biological fluids via hydrolysis of the silica matrix into orthosilicic acid, which is then primarily excreted via renal clearance [140].

Calcium phosphate (CaP) nanocarriers have also proven promising for pulmonary transepithelial delivery. Inhalation of therapeutics has been an administration route of interest for the treatment of cardiac conditions. Following respiration, blood oxygenated in the alveoli of the lungs travels by the pulmonary vein to the heart. Miragoli and colleagues investigated the translocation of calcium phosphate nanoparticles loaded with therapeutic peptide, R7W-MP, from inhalation to the site of treatment in the heart for the treatment of diabetic cardiomyopathy. The group utilized CaP nanoparticles, which were 20–50 nm in diameter, biocompatible, biodegradable, and negatively charged. The selected negative charge provided protection from enzymatic degradation and enhanced cardiomyocyte cellular permeability. The group noted that the CaP particles did not elicit cardiotoxic effects as observed with other inorganic nanoparticles such as TiO_2_, SiO_2_, and Co_3_O_4_ derived particles, as well as did not elevate immune markers. A therapeutic effect was observed in mice and pigs [151].

### 6.3. Nanoliposomes and Nanomicelles

Nanoliposomes and nanomicelles, as discussed previously, are highly biocompatible due to their phospholipid-based structure. In addition to biocompatibility, the phospholipid structure of liposomes is favorable for interaction with pulmonary surfactants. However, liposomes are frequently cleared by macrophages [148]. Surface modification of liposomes can be implemented to achieve drug delivery goals. As examples, modifications such as chitosan coating [152] or incorporation of polyvinyl alcohol with a hydrophobic anchor [153] can be used to increase retention to prolong therapeutic delivery. Liposomes modified with oligosaccharide chitosan were observed to reversibly open tight junctions in Calu-3 cells, a human lung epithelial cell line, as measured by TER, to enhance delivery [153].

Recently, Yu et al. modified liposomes functionalized with an Fc receptor ligand and compared particle stiffness for the delivery of dexamethasone [154]. Particle functionalization with Fc receptors has previously been observed to enhance transcytosis in Calu-3 cells [155]. To create a stiff liposome, a PLGA nanoparticle was encapsulated as a core of a liposome. The group observed that the stiff nanoparticles demonstrate greater bronchial mucosal uptake in rat lungs in comparison to soft particles, as well as increased endocytosis and exocytosis in Calu-3 cells. Treatment with stiff particles was associated with increased actin filament aggregation [156]. The group also demonstrated success using this platform to treat airway inflammation as it occurs in asthma [154].

## 7. Oral Drug Delivery

Oral delivery of therapeutics is widely considered the preferable route of delivery by patients. Oral delivery is non-invasive, allowing for convenient and comfortable administration of therapeutics. Oral delivery options have also been identified to increase adherence to prescribed drug protocols and dosing schedules and can allow for more flexible fabrication requirements. While oral delivery is very effective for small molecule therapeutics, bioavailability is severely limited for other therapeutic classes, such as proteins and peptides. The stomach subjects oral dosage forms to a highly acidic and enzyme-rich environment prior to reaching the small intestines as the primary site of absorption. Thus, orally delivered therapeutics also need to be designed to be protected from the low pH of the stomach and to release their therapeutic agents in the elevated pH of the small intestine [157].

**Figure 7:**
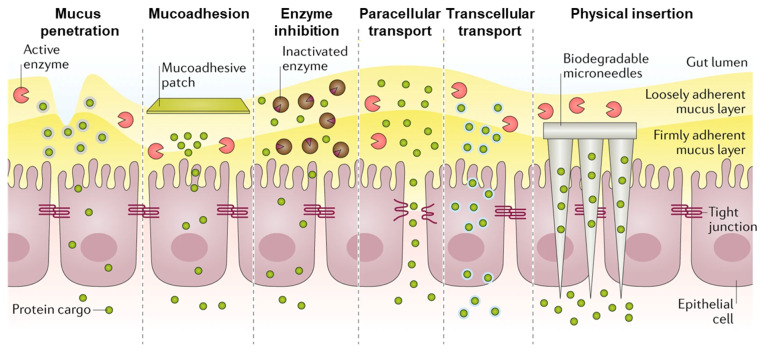
Common approaches that have been used to achieve oral drug delivery. These include mucus penetration, cohesion, enzyme inhibition, opening up of paracellular transport, facilitation of transcellular transport, and physical insertion. Mucus-penetrating coatings facilitate the transit of proteins and peptides through the loosely adherent and firmly adherent mucus layers. Mucoadhesive polymer coatings increase the drug residence time at the desired site, reducing dilution effects. Protease inhibitors inactivate proteolytic enzymes found in the digestive tract to prevent protein degradation. Paracellular permeation enhancers transiently disrupt tight junction complexes between adjacent epithelial cells through events such as calcium chelation or modulation of intracellular signaling cascades. Transcellular permeation enhancers enable the translocation of the protein cargo by facilitating its diffusion through the cell. Physical insertion methods pierce the intestinal lining and directly administer a protein payload to the underlying vasculature. From [162] used with permission.

The intestinal epithelium consists of several cell types with distinct functions (Figure 7). This includes enterocytes, Goblet cells, enteroendocrine cells, Paneth cells, microfold cells, cup cells, and tuft cells [158]. Enterocytes and goblet cells are essential due to their role in absorption and mucus secretion, respectively [159,160]. In order to maintain tissue homeostasis, the intestinal epithelium allows for selective permeability of solutes while restricting the passage of pathogens, antigens, and other nondesirable materials. Generally, diffusion across intestinal epithelial tissues is limited to molecules less than 700 Da, and paracellular permeability is limited to hydrophilic molecules <200 Da [161].

Enterocytes are the primary target for nanoparticle transport as they make up 90–95% of cells lining the GI tract [163]. Various properties can affect the ability of nanoparticles to make their way through enterocytes. These include particle size, materials, and surface chemistry. Enterocytes preferentially internalize and transport nanoparticles of 20–100 nm in diameter [145]. PAMAM can be transported across intestinal epithelial barriers in rats and human cells [163,164]. Enterocytes that transport nanoparticles to lysosomes will degrade them, necessitating the need to design particles away from the degradative pathway and towards the transcytotic pathway.

As is the case in the lung, mucus is a major barrier to the absorption of orally administered particles [165]. Mucus is continuously secreted to remove pathogens by trapping foreign materials and rapidly clearing them. Nanostructure size and surface properties are critical engineering parameters for designing materials to overcome the mucus layer. Neutrally charged or zwitterionic materials reduce charge-based interactions with mucins, size can be selected to be appropriate to the porosity of the mucus of interest, and proteolytic enzymes may be incorporated into particle design. The incorporation of mucoadhesive properties can be used to extend particle residence time and increase drug release in close proximity to the epithelial surface [157]. Considerations for design [166] and recent advances in mucus-penetrative drug carriers have been recently reviewed elsewhere [167,168].

### 7.1. Particle Geometry and Physical Characteristics

Epithelial uptake of nanoparticles is dependent upon many aspects of nanoparticle morphology and surface characteristics. Decreased particle sizes, such as 50 nm or 200 nm diameters, have been found to increase particle uptake and transepithelial transport of polystyrene nanoparticles in comparison to 500 and 1000 nm particles in Caco-2 cell culture models [169]. Size has also been identified to alter particle behavior traversing epithelial monolayers [170]. Particle shape has also been found to impact transport, with similar volume biotin-conjugated polystyrene rods demonstrating the greatest transport, followed by discs, then spheres of the same material [169]. Orally administered particles have comparable shape-dependent pharmacokinetics in vivo as well [171].

Shape-based effects on cell uptake have also been explored, identifying mesoporous silica nanorods as a platform that enables increased cellular uptake in comparison to mesoporous silica nanospheres [172]. Each form factor was found to be internalized by different pathways of endocytosis; nanorod endocytosis involved the caveolae-dependent pathway, whereas nanospheres involved clathrin-dependent endocytosis [172]. In the further evaluation of the effect of shape and surface topology, Wang et al. demonstrated that chiral mesoporous silica nanoscrews enhanced cellular uptake, bioadhesion, and mucus penetration in comparison to chiral mesoporous silica nanorods and chiral mesoporous silica nanospheres [173].

In addition to size and surface charge, particle stiffness or elasticity has also been identified as a particle parameter that influences interaction with the epithelium [174,175]. While influential, findings in this space are mixed. Yu et al. examined the effect of particle rigidity of PLGA nanoparticles on transcytosis [174]. The group found that unmodified, stiff nanoparticles demonstrated higher transcytosis efficiency in comparison to soft nanoparticles. However, when the group decorated the soft and stiff particles with FcBP, an Fc receptor domain binding peptide, soft nanoparticles were more effectively transcytosed than stiff nanoparticles [174]. This demonstrated that receptor binding and particle stiffness are two dependent parameters that can be altered to control nanoparticle targeting.

Zheng et al. also explored the effect of elasticity on nanoparticle transcytosis and enhanced insulin delivery using zwitterionic hydrogel nanoparticles with adaptable elasticity [175]. They identified that an increase in elasticity increased transcytosis, increased in vivo bioavailability of insulin, as well as altered intracellular trafficking of endocytosed nanoparticles. The results suggested that higher elasticity nanoparticles were more likely to reach secretion-related pathways than degradation-related, such as late endosomes or lysosomes [175].

Polymeric nanoparticles can improve transport across the mucosal barrier. The flexible design of polymeric nanoparticles allows for controlled characteristics, such as size, surface chemistry, and targeting [176]. Polymeric nanoparticles can also be designed to protect therapeutic cargo from environmental degradation in the GI tract and allow for multiple formats of drug loading. For instance, drugs can be loaded into the core of the particle, conjugated to the particle surface, or conjugated to a polymer subunit in the particle design. These choices can be used to maintain therapeutic stability or facilitate the loading of therapeutics of different characteristics, such as accommodating molecular weight or hydrophobicity, or to engineer various release mechanisms [145]. Common synthetic materials for nanoparticle fabrication include poly(lactic acids), poly(lactic-co-glycolic acids), poly(ɛ-caprolactone), poly(methyl methacrylates), and poly(alkyl cyanoacrylates) [176,177].

### 7.2. Inorganic Nanoparticles

Inorganic nanoparticles have also been investigated for oral transepithelial delivery. Lamson et al. explored the parameter space of silica nanoparticles and their interaction with the intestinal epithelia. They identified that smaller and more negatively charged nanoparticles increased drug permeation in vitro and in vivo with co-delivery of insulin that was able to control serum glucose levels [178]. The silica nanoparticles were identified to modulate barrier function via an integrin-dependent and MLCK-dependent mechanism. Silica nanoparticles were found to act apically and did not cross the epithelial barrier, instead serving as facilitators of transepithelial insulin delivery [178].

Citrate-capped gold nanoparticles have also been found to increase paracellular permeability reversibly, inducing changes to CLDN1 and ZO-1 in Caco-2 cells [179]. Hydrophilic, electrically neutral mesoporous nanoparticles have been found to traverse the mucus and be transcytosed in Caco-2 cells via the caveolae-mediated pathway, facilitating clinically significant delivery of insulin. These particles were designed using structural principles used by viruses, which are decorated with both positively and negatively charged amino acids in order to pass through both mucus and epithelium [180].

### 7.3. Chitosan and Polymer Derivatized Nanoparticles

Surface properties of nanoparticles are a key property determining uptake by intestinal epithelial cells. PEG is a common nanoparticle coating because PEGylation can help protect the nanoparticle surface from enzymatic degradation and can extend circulation time by preventing rapid clearance by the kidneys due to its stabilizing properties [145,176]. The most used natural polymers in nanoparticles are sodium alginate, albumin, chitosan, and gelatin [176,177,181]. Chitosan is the most widely used natural polymer in nanoparticles. Chitosan’s popularity is largely due to properties of permeation enhancement and mucoadhesion that are particularly useful for epithelial delivery [182,183]. The hydrophilicity of both PEG and chitosan also enhances transport across the intestinal mucosa [176].

Chitosan has been identified to enhance paracellular permeability via electrostatic interactions with apical integrins, initiating a signaling cascade involving phosphorylation of FAK and Src tyrosine kinases and ultimately leading to a shift in CLDN4 localization to the cytosol [184,185]. Chitosan depolymerizes cellular F-actin associated with the tight junction protein ZO-1 [186]; increasing tight junction permeability has the potential to allow all content in the intestinal tract access to the basal pole. Consistent with this, analysis of oral drug delivery has shown that significantly higher amounts of macromolecular drugs can be transported after co-administration with chitosan [187].

As a result of its favorable properties, chitosan has been used to create chitosan particles as well as used as a surface modification of other particle systems, such as trimethyl chitosan chloride-coated insulin-loaded PLGA nanoparticles. These chitosan-coated nanoparticles demonstrated improved cellular uptake via clathrin- or adsorption-mediated endocytosis as well as permeation via tight junction opening, in comparison to unmodified PLGA nanoparticles [188]. Continued work to chemically modify chitosans has yielded particles with increased capacity to open tight junctions, such as with mercaptonicotinic acid activated-thiolated chitosan nanoparticles used to enhance oral peptide delivery [189].

Another recent example uses fluorocarbon-modified chitosan to create nanocomplexes with therapeutic antibodies contained within an enteric capsule. The nanocomplexes induced tight junction rearrangement and achieved an in vivo response comparable to intravenous delivery [190]. Polymeric systems can be designed to be microenvironment-adaptive to accommodate the requirements to traverse both the mucosal and cellular epithelial barriers. Such systems include polymeric particles using PLGA-hydrozone-PEG copolymer to facilitate a hydrophilic–hydrophobic switch at the slightly acidic epithelial surface [191].

### 7.4. Targeted Nanoparticles and Dendrimers

Nanoparticles can be functionalized with targeting peptides to improve therapeutic delivery. Tight junction-modulating peptides have also been used to decorate nanoscale systems to enhance transepithelial therapeutic delivery. For example, Lee et al. examined the use of chitosan and AT-1002, a peptide derived from zonula occludins toxin, as nanocarrier modifications in order to enhance transepithetlial delivery. The group identified that either chitosan or AT-1002 functionalized nanocarriers independently decreased TER, FITC-insulin permeation and improved in vivo blood glucose response, but dual conjugation with both AT-1002 and chitosan yielded a greater effect [192]. Other tight junction-disrupting peptides have been used in blood–brain barrier work [28]. FcRn-targeted PLGA-PEG nanoparticles leverage FcRn-mediated transcytosis to enhance transepithelial delivery of semiglutide in human intestinal organoids [193].

Recently, a ligand-switchable PLGA nanoparticle system was developed by Yang et al. to facilitate multistep targeting for orally delivered insulin. The nanoparticle is designed with cell-penetrating peptide (Pep) conjugated to the particle via a pH-triggered stretch element and a galactose (Gal). In an acidic environment, the Pep is stretched to facilitate transepithelial delivery, and following transcytosis, at the pH of circulation, the stretchable element folds to expose the Gal for hepatic targeting [194].

PAMAM dendrimers have also been explored for transepithelial therapeutic delivery. In Caco-2 cells, Kitchens et al. [195] demonstrated permeability of mannitol, decreases in TER, and alterations to occludin and actin under treatment with multiple categories of PAMAM dendrimers, suggesting the interaction of the PAMAM dendrimers causing the opening of the tight junctions. The group noticed an increase mannitol permeability associated with an increase in the size of anionic dendrimers evaluated in comparison to smaller anionic dendrimers [195]. However, an increase in dendrimer generation number or size has also been found to increase cellular toxicity [196]. Continued work identified that dendrimer composition choices, such as using either ester-linked glycine or beta-alanine spacer linker chemistries to attach drug, could alter the transepithelial pathway dominant in the transport of the dendrimer [197].

### 7.5. Permeation Enhancers

Many macro- and microscale drug delivery platforms have been developed for oral delivery of biologics to the luminal environment. These platforms include enteric capsules, tablets, and hydrogels, designed to protect a therapeutic through the GI tract and control release. However, many require the use of additional methods for therapeutic to traverse the mucus and epithelial layers to achieve sufficient bioavailability. These obstacles have motivated groups to develop permeation enhancers with more refined mechanisms of action and timelines of impact [28]. For instance, oral devices, like the self-orienting millimeter-scale applicator (SOMA) and luminal unfolding microneedle injector (LUMI), have used injection to bypass the barriers of GI mucosa [198,199].

A more flexible approach is to include the co-delivery of chemical permeation enhancers with biologic therapy via incorporation into the therapeutic formulation. However, the use of chemical permeation enhancers raises concerns over the often limited functional increase in bioavailability and toxicity deriving from long-term use [199]. Early-developed permeation enhancers have broad mechanisms of action and result in extended disruption of cellular function [28].

More recently, Lamson and colleagues screened a library of plant-based compounds for the capacity to facilitate protein permeation and identified a strawberry-derived permeation enhancer, pelargonidin, that induces reversible alterations to tight junctions [200,201]. When orally co-administered with insulin, pelargonidin was able to lower serum glucose levels for over 4 h, indicating physiologic efficacy.

As another approach to improve the absorption of biologics, nanostructured films (NSFs) are being studied as a means to increase transepithelial delivery. Nanostructured films fabricated with either polypropylene (PP) or polyether ether ketone (PEEK) were used to treat gastrointestinal epithelial cells [36,202]. Kam et al. demonstrated that the application of NSFs to model epithelial monolayers induced an increase in permeation of FITC-BSA, FITC-IgG, and Etanercept. They also found application of NSFs induced a decrease in barrier function, which was reversible upon removal of the film [202]. Work by Stewart et al. showed that treatment with the NSFs made direct contact with the apical surface of Caco-2 cells. The treatment with the NSFs showed that there was a decrease in barrier function after treatment. The NSFs specifically affected the transcytosis pathway and concurrently also affected the paracellular pathway [36]. Further work examining the effect of NSF on the paracellular pathway, conducted by Huang et al., identified that the application of the NSFs induced dynamic remodeling of scaffold protein ZO-1, in which junction-associated ZO-1 was displaced into cytosolic complexes. The cytosolic ZO-1 complexes were observed to colocalize with claudin proteins and associate with F-actin during formation and circulation through the cytosol. The remodeling rate of ZO-1 was found to be increased under treatment with NSFs, identified via measurement of fluorescence recovery after photobleaching (FRAP) [67]. Pairing nanostructured films with therapeutics that demonstrate limited oral bioavailability, such as biologics, or decorating the NSFs with therapeutics, such as antibiotics, could be applied to treat a variety of diseases and conditions.

## 8. Conclusions and Future Directions

Successful drug delivery requires careful consideration of the requirements and constraints of the therapeutic and target to inform drug carrier design. Such considerations include the route of administration, barriers encountered, the chemical characteristics and class of therapeutic, and whether the delivery is intended to be local or systemic (Table 1).

While ultimate targets of treatment vary when considering noninjectable delivery strategies, the need to traverse epithelial barriers is a recurrent obstacle across many organ systems. Aspects of transepithelial delivery vary by organ system, such as the consideration of multiple dermal layers in the skin, mucus in the intestine, or surfactant in the lung, but many fundamentals of transepithelial delivery remain unchanged across organ systems. Regardless of organ system, the feasibility of transcytosis or paracellular transit of a therapeutic molecule must be identified and, in many cases, facilitated by additional technologies to promote the passage of pharmacologically active agents.

To date, most approaches targeting the paracellular route have focused on targeting bicellular junctions. Tricellular junctions represent an intriguing alternative with a unique composition that has the potential to be specifically targeted. Of note, tricellular junctions have more permissive permeability characteristics that could be leveraged for the delivery of molecules or nanoparticles that are too large to go through the bicellular route.

Nanoscale materials, whether they be particles, architectures, or otherwise, present an ever-expanding toolkit for the development of drug delivery vehicles. The variety of fabrication methods and materials available facilitate refined control of key physical and chemical properties, such as size, charge, and availability of functional groups for conjugation. In addition to facilitating transepithelial transit, these properties can improve other aspects of therapeutic delivery, such as supplying further drug targeting or enabling controlled or prolonged therapeutic release.

Designing agents to facilitate transepithelial delivery is not a “one size fits all” approach. Rather, the integration of understanding epithelial biology with innovative biomaterial formulations within the context of treatment goals and organ systems will facilitate the most effective solutions. Innovation bridging these fields will continue to expand the feasibility of additional treatment routes for a variety of therapeutics. Ultimately, this expansion will provide greater flexibility in tailoring the most effective modes of treatment delivery to improve patient outcomes.

## Figures and Tables

**Table 1:** Nanomaterial systems observed to modify transepithelial transit or barrier function.

Categories	Material	Barrier	Reduced Results Related to Transepithelial Transit or Barrier Function
Nanoscale modifications of bulk material	Nanostructured surfaces: polypropylene, PEEK	Dermal	Reversible enhanced permeation, tight junction (TJ) rearrangement, and actin cytoskeleton rearrangement [64,65,66]
		Oral	Reversible enhanced permeation, TJ rearrangement, actin cytoskeleton rearrangement, transcytosis, and paracellular enhancement [36,67,202]
	Nanoporosity	Ocular	Prolonged and controlled topical release [203]
Inorganic nanoparticles (NP)	Silica NP	Oral	Smaller and more negatively charged NP increased drug permeation and modulated barrier function [178]
	Mesoporous silica NP	Oral	Shape impacted uptake, internalization [172], and adhesion [173], virus-inspired hydrophilic, neutrally charged NP transited mucus and transcytosed barrier [180]
		Pulmonary	Surface modification with PEI and PEG facilitated reach of distal lungs and alleviation of inflammatory response [149]
	Gold NP	Dermal	Size-dependent transdermal permeation [78,79], charge-modified permeation [80,81], and shape-modified permeation [82]
		Oral	Citrate-capped gold NP reversibly increased paracellular permeation [179]
	Calcium phosphate NP	Pulmonary	Transcytosis through lung epithelium and successful cardiac targeting [151]
	Chitosan ceria NP	Ocular	Led to disruption of TJs and drug permeation [120]
	Chitosan meso-porous silica NP	Dermal	Paired with chemical permeation enhancers [85] or composite system such as a gel [86] to facilitate skin permeation
Polymeric NP and complexes	Chitosan NP, including nanocomplexes and nanomicelles	Dermal	Delivery of multiple classes of drug cargos, reversible drop in TER, paracellular delivery, and opening of TJs [88]
		Oral	Permeation enhancement [182,183,187], mucoadhesion [182,183], enhanced transport [176], enhanced paracellular permeability [184,185,186,189], and TJ rearrangement [190]
		Ocular	Zwitterionic chitosan nanocomplexes transiently opened TJs, delivery of high molecular weight therapeutics to the retina and choroid [103,119]
	Polystyrene NP	Oral	Particle size modified particle uptake [169] and transit [170], particle shape altered transit [169,171]
	PLGA NP	Oral	Particle stiffness and receptor binding altered transcytosis [174], targeting can be further refined such as ligand switchable system [194]
	Zwitterionic hydrogel NP	Oral	Increase in elasticity increased transcytosis, bioavailability of insulin, and increased likelihood of secretion rather than degradation pathways [175]
	PEG surface modification of polymeric NP	Oral	Increases intestinal epithelial cell uptake [145,176], hydrophilicity enhances transport
		Ocular	Nanomicelles of PEG, poly(propylene glycol), and poly(ɛ-caprolactone) successful retinal delivery likely via transcorneal transcytosis [117]
	Chitosan surface modification of polymeric NP	Oral	Enhanced permeation, mucoadhesion [182,183], hydrophilicity enhances transport [176] and NP uptake [188], enhanced paracellular permeability [184,185,188]
		Ocular	Surface modification with chitosan and peptide transporter-1 targeting elements facilitates transit to the posterior region of the eye [115,116]
	Cationic gelatin NP	Ocular	Plasmid delivery via particles restored corneal epithelial barrier integrity [118]
	PAMAM dendrimers	Oral	Disrupted TJs, increased drug permeability associated with size increase and charged dendrimers [195], dendrimer composition altered transepithelial path [197]
Lipid-based NP	Liposomes	Dermal	Modified liposomes, such as niosomes, reached the epidermis and the dermis [89,90]
		Pulmonary	Coating with chitosan or hydrophobic anchors prolonged retention, opened TJs, and enhanced paracellular delivery [152,153]. Fc receptor functionalization increased transcytosis [154,155], increased stiffness, and increased endo and exocytosis [154].
		Ocular	PAMAM dendrimer-coated liposome demonstrated transcorneal permeability and posterior chamber therapeutic response [121,122], observed to transit via the transcellular and paracellular routes with disruption of TJs [122]
	SLNs and NLCs	Dermal	Increased permeation by creating occlusive film at surface, enhancing hydration [92]
	Lipid nanocapsules	Ocular	cRGD decorated nanocapsules traversed the choroidal endothelial barrier and the retinal pigment epithelial barrier to achieve therapeutic results in retina [123]
